# Sinus Arrest in Asymptomatic COVID-19 Infection

**DOI:** 10.7759/cureus.23736

**Published:** 2022-04-01

**Authors:** Alexander Nguyen, Jessica Corcoran, Christopher D Nedzlek

**Affiliations:** 1 Department of Emergency Medicine, Henry Ford Health, Wyandotte, USA

**Keywords:** sinus pause, sinus node dysfunction, syncope, bradycardia, sinus arrest, covid-19

## Abstract

Coronavirus disease 2019 (COVID-19) is a viral respiratory tract syndrome capable of affecting a multitude of major organs in the human body. It is a known cause of severe vascular compromise, myocardial ischemia, myocarditis, and various cardiac dysrhythmias. Dysfunction of the sinoatrial (SA) node, the primary pacemaker of the heart, can arise from structural heart disease, medications, electrolyte abnormalities, and hypothyroidism. We report and discuss a case of a 50-year-old female with no significant past medical history (PMH) and no SA dysfunction risk factors, who experienced multiple syncopal events and an episode of sinus arrest characterized by transient asystole captured with telemetry monitoring. The patient was incidentally found to be COVID-19 positive and displayed no signs or symptoms concerning the viral illness. Despite our patient’s lack of respiratory issues or other symptomatology, a significant and potentially fatal relationship exists between her viral infection and cardiac sequelae.

## Introduction

The sinoatrial (SA) node serves as the dominant pacemaker of the heart, and its dysfunction leads to clinically significant syncope and hypotension [1]. Etiologies for possible SA node dysfunction include, but are not limited to, infiltrative disease, infection, genetic mutations, ischemia, medications, electrolyte derangements, and thyroid abnormalities [1].

Coronavirus disease 2019 (COVID-19) is a respiratory illness capable of damaging several organ systems in the human body, particularly the heart. Cardiovascular complications include ischemia, myocarditis, heart failure, and dysrhythmia [2]. Newly unveiled reports in the medical literature implicate COVID-19 in cardiac conduction system abnormalities, specifically of the SA node and atrioventricular (AV) node. While the exact mechanism remains unclear, it is thought to be the result of hypoxemia, inflammation, or direct viral infiltration leading to myocardial compromise [3].

## Case presentation

We report and discuss a case of a 50-year-old female with no significant past medical history (PMH), who presented to the emergency department (ED) after experiencing a syncopal event at home. She reports syncopizing from a standing position leading to subsequent head trauma. She denied a prodrome prior to the event as well as other symptoms, including chest pain or dyspnea.

She had received no COVID-19 vaccinations and took no medications or illicit drugs, was a lifetime non-smoker, and had no known allergies. On physical exam, she was well-appearing and in no acute distress. She had a small left frontal contusion with no abrasions or active bleeding. She was alert and oriented to person, place, and time with no focal neurologic deficits and no motor, sensory, or speech changes. Initial vital signs were unremarkable-blood pressure: 126/73 mmHg, heart rate (HR): 93 beats per minute (bpm), respiratory rate: 16 breaths per minute, oxygen saturation 100% on room air, and afebrile. An electrocardiogram (ECG) demonstrated normal sinus rhythm (NSR) with no abnormal intervals, T-wave changes, or signs of ischemia.

During the evaluation, the patient became acutely unresponsive and experienced an episode of abrupt bradycardia in which her HR began to precipitously decline. She was immediately evaluated for a central pulse, which was present upon palpation-during which simultaneous normalization of her HR to 70 bpm occurred. Chest compressions were not initiated and hypoxia was not observed. The patient remained hemodynamically stable after the event and had no recollection of what occurred. A repeat ECG was obtained which continued to show NSR with no signs of ischemia or other abnormalities.

**Figure 1:**
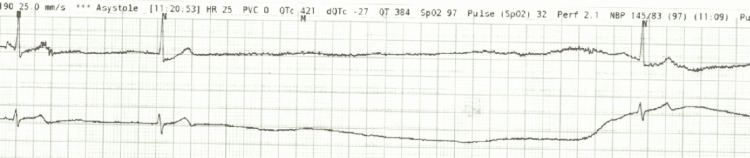
Episode of asystole suspicious for sinus arrest captured on telemetry monitoring

Subsequent laboratory workup demonstrated no abnormalities-negative high sensitivity troponin and no electrolyte derangements, lactic acidosis, leukocytosis, or anemia. N-terminal pro-B-type natriuretic peptide (BNP), thyroid-stimulated hormone (TSH), procalcitonin, venous blood gas (VBG), inflammatory markers, and acute phase reactants were within normal limits (WNL). Quantitative D-Dimer resulted in a value less than the reference range of <0.50 μg/mL. Pregnancy testing was negative and urinalysis was negative for infection. The patient was incidentally found to be COVID-19 positive, further corroborated by a chest radiograph which showed bibasilar interstitial opacities. Despite this, the patient denied viral symptomatology or other complaints. A rhythm strip was obtained which successfully captured the adverse cardiac event during telemetry monitoring (Figure 1). It showed a five-second asystolic event before the abrupt resumption of NSR-findings concerning sinus arrest.

**Figure 2:**
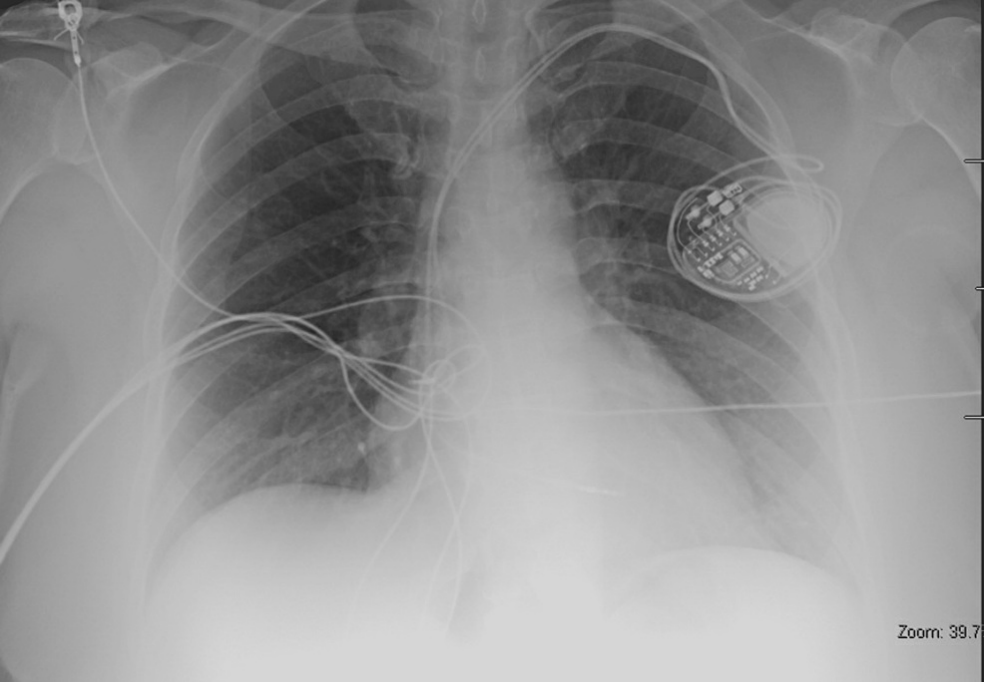
Chest radiograph demonstrating adequate cardiac pacemaker placement

Cardiology was consulted and advised no acute interventions as the patient remained hemodynamically stable. The patient was asked to follow nothing by mouth with plans to place a cardiac pacemaker the following morning. In the interim, central venous access was established and transcutaneous pacer pads were placed on the patient in the event of a subsequent episode. The patient was closely monitored in the intensive care unit (ICU) overnight with no additional adverse events. A transthoracic echocardiogram revealed a normal left ventricular ejection fraction of 59% and an unremarkable examination as a whole. The following morning, a dual-chamber permanent pacemaker under cardiac fluoroscopy was implanted without issue (Figure 2).

The patient’s level of care was de-escalated to the general medical floor with telemetry monitoring. Steroids and other antiviral medications were not indicated as the patient remained normoxic throughout her hospital stay. The patient was medically cleared and discharged home on hospital day three with outpatient primary care and cardiology follow-up as well as with a referral for infusion of COVID-19 monoclonal antibodies.

## Discussion

Cardiac dysrhythmias in the setting of active COVID-19 infection are believed to occur due to a variety of mechanisms, including direct cardiac myocyte injury, hypoxemia leading to cardiac cell death, myocardial inflammation, and inflammatory cytokine burden [4]. Despite these possible etiologies, the exact mechanism remains unclear.

A report in the medical literature describes multiple episodes of sinus arrest in the setting of COVID-19 infection. The patients in this study were hypoxemic and experienced profound bradycardia and sinus pauses with increasing frequency as their oxygen requirements and needs for respiratory support escalated [2]. Their bradyarrhythmia would ultimately resolve without the need for specific intervention as the infection and their hemodynamic status improved [2]. This suggests a correlation between bradyarrhythmia and COVID-19 infection.

Moreover, SA node dysfunction has been seen in patients with active, stable COVID-19 infection who were asymptomatic from a cardiac standpoint. These patients were successfully managed conservatively without the need for medication, electricity, or invasive modalities. Although their respiratory status was stable and they did not require supplemental oxygen, they were nonetheless symptomatic from a viral standpoint with upper respiratory symptoms such as cough, dyspnea, and nasal congestion [5].

An additional report describes episodes of asystole in a patient who had previously tested positive for COVID-19 and denied viral symptomatology. The patient’s laboratory workup was significant for leukocytosis, lactic acidosis, and elevated creatinine kinase which would indicate a possible ongoing systemic or inflammatory response [6]. Similarly, our patient experienced two episodes of syncope and subsequent transient sinus arrest in the setting of completely asymptomatic COVID-19 infection without systemic illness. She possessed no significant PMH or cardiovascular risk factors. Moreover, her family and social history were non-contributory and she was never hypoxemic. To our knowledge, there are no reports of SA node dysfunction as the sole clinical manifestation in a completely asymptomatic patient who was also without other surrogate markers of infection or systemic signs only incidentally found to be COVID-19 positive. Nonetheless, very few reports in the medical literature describe SA node dysfunction in stable COVID-19 patients with no history or risk factors for cardiac disease.

It is for all of these reasons that patients infected by COVID-19 with or without cardiovascular or respiratory compromise should closely monitor their symptoms as they are potentially at risk for acute decompensation. Clinicians must maintain a high index of suspicion for bradyarrhythmia and SA node dysfunction in patients presenting with syncope or other anginal equivalents. Furthermore, COVID-19 infection must be on the differential diagnosis for patients experiencing clinically significant cardiac manifestations, including sinus arrest, regardless of viral symptomatology. At this time, it is unknown whether COVID-19 increases the risk of recurrent sinus arrests. As such, further studies are unequivocally needed.

## Conclusions

COVID-19 is well known to affect a variety of organ systems in the human body, in particular, the cardiopulmonary and vascular systems. The virus is capable of damaging the electrical conduction system of the heart both directly from viral infiltration and indirectly through hypoxemia and inflammation. Once thought to be a sequela of moderate to severe viral illness, our report suggests that COVID-19 is capable of deleterious cardiac manifestations in completely asymptomatic persons who are without systemic illness or physiologic derangements. As new COVID-19 clinical manifestations continue to be unearthed, it is critically important that the clinician maintain a high index of suspicion for this phenomenon and other cardiac sequelae, so that time-sensitive treatment and management are expeditiously employed.

## References

[1] Adán V, Crown LA (2003). Diagnosis and treatment of sick sinus syndrome. Am Fam Physician.

[2] Olagunju A, Forst B, Yakymovych O, Yeneneh BT (2021). Multiple sinus pauses in a patient with COVID-19. Cureus.

[3] Peigh G, Leya MV, Baman JR, Cantey EP, Knight BP, Flaherty JD (2020). Novel coronavirus 19 (COVID-19) associated sinus node dysfunction: a case series. Eur Heart J Case Rep.

[4] Wang Y, Wang Z, Tse G (2020). Cardiac arrhythmias in patients with COVID-19. J Arrhythm.

[5] Babapoor-Farrokhran S, Batnyam U, Wiener PC, Kanjanahattakij N, Khraisha O, Amanullah A, Mainigi SK (2020). Atrioventricular and sinus node dysfunction in stable COVID-19 patients. SN Compr Clin Med.

[6] Needleman JS, Anderson WL, Gray MT, Tanawuttiwat T, Bateman PV (2022). Asystole in a COVID-19 patient without systemic illness: a case report. Oxf Med Case Reports.

